# Comparative transcriptomic and proteomic signature of lung alveolar macrophages reveals the integrin CD11b as a regulatory hub during pneumococcal pneumonia infection

**DOI:** 10.3389/fimmu.2023.1227191

**Published:** 2023-09-18

**Authors:** Kristina Zec, Stephanie Thiebes, Jenny Bottek, Devon Siemes, Philippa Spangenberg, Duc Viet Trieu, Nils Kirstein, Nirojah Subramaniam, Robin Christ, Diana Klein, Verena Jendrossek, Maria Loose, Florian Wagenlehner, Jadwiga Jablonska, Thilo Bracht, Barbara Sitek, Bettina Budeus, Ludger Klein-Hitpass, Dirk Theegarten, Olga Shevchuk, Daniel R. Engel

**Affiliations:** ^1^ Institute for Experimental Immunology and Imaging, Department of Immunodynamics, University Hospital Essen, Essen, Germany; ^2^ Kennedy Institute of Rheumatology, University of Oxford, Oxford, United Kingdom; ^3^ Institute for Cell Biology (Cancer Research), University Hospital Essen, Essen, Germany; ^4^ Clinic for Urology, Paediatric Urology and Andrology, Justus-Liebig University of Giessen, Giessen, Germany; ^5^ Department of Otorhinolaryngology, University Hospital Essen, University Duisburg-Essen, Essen, Germany; ^6^ Medical Faculty, Medizinisches Proteom‐Center, Ruhr‐University Bochum, Bochum, Germany; ^7^ Clinic for Anesthesiology, Intensive Care Medicine and Pain Therapy, University Hospital Knappschafts-krankenhaus Bochum, Bochum, Germany; ^8^ Institute of Cell Biology (Cancer Research), Faculty of Medicine, University of Duisburg-Essen, Essen, Germany; ^9^ Institute of Pathology, University Hospital Essen, Essen, Germany

**Keywords:** alveolar macrophage, proteomics, bioinformatics, transcriptomics, infection

## Abstract

**Introduction:**

*Streptococcus pneumoniae* is one of the main causes of community-acquired infections in the lung alveoli in children and the elderly. Alveolar macrophages (AM) patrol alveoli in homeostasis and under infectious conditions. However, the molecular adaptations of AM upon infections with *Streptococcus pneumoniae* are incompletely resolved.

**Methods:**

We used a comparative transcriptomic and proteomic approach to provide novel insights into the cellular mechanism that changes the molecular signature of AM during lung infections. Using a tandem mass spectrometry approach to murine cell-sorted AM, we revealed significant proteomic changes upon lung infection with *Streptococcus pneumoniae.*

**Results:**

AM showed a strong neutrophil-associated proteomic signature, such as expression of CD11b, MPO, neutrophil gelatinases, and elastases, which was associated with phagocytosis of recruited neutrophils. Transcriptomic analysis indicated intrinsic expression of CD11b by AM. Moreover, comparative transcriptomic and proteomic profiling identified CD11b as the central molecular hub in AM, which influenced neutrophil recruitment, activation, and migration.

**Discussion:**

In conclusion, our study provides novel insights into the intrinsic molecular adaptations of AM upon lung infection with *Streptococcus pneumoniae* and reveals profound alterations critical for effective antimicrobial immunity.

## Graphical Abstract

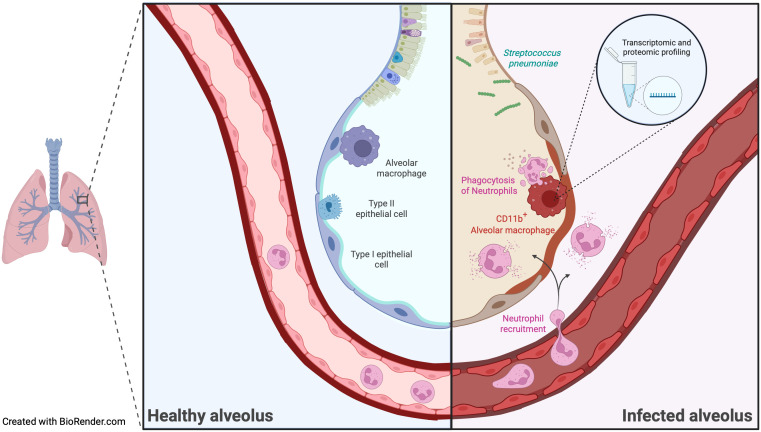

## Introduction

Bacterial respiratory infections are mainly caused by the gram-positive bacterium *Streptococcus pneumoniae* (*S. pneumoniae*) (1). Children, elderly patients, and individuals with certain underlying conditions, such as immunosuppressive states, are at increased risk of developing an invasive and severe disease (2). Alveolar macrophages (AM) are lung-resident cells that perform critical functions and represent the first line of defense against respiratory pathogens. Immunosuppressive interactions between AM and the alveolar epithelium maintain AM in a quiescent state (3, 4). Upon infection, ligation of several activating receptors, such as Toll-like receptors (TLRs) and cytokine receptors, overrides the inhibition and results in AM activation to clear the pathogen (5, 6). Activated AM exhibits enhanced phagocytosis, reactive oxygen species (ROS), and cytokine production (7). Importantly, AM must coordinate their response to ensure efficient clearance of pathogens while limiting excessive inflammation that could damage the alveoli and impair lung function. Among other important functions, AM phagocytose *S. pneumoniae* and recruit neutrophils to the site of infection (8, 9). Subsequently, AM also become important in removing apoptotic neutrophils (10), but the molecular adaptations during infections with *S. pneumoniae* are incompletely understood.

CD11b (also known as ITGAM) is a cell surface protein that is present in various immune cells. AM express only low levels of this integrin in homeostasis (3), but increased expression has been observed under inflammatory conditions and in infections with *S. pneumoniae* (11, 12). Regarding bacterial lung infections, CD11b-deficient mice showed increased infection strength after challenge with *S. pneumoniae* (13), suggesting a critical role of CD11b expression by AM. However, further studies are required to study the cell-specific regulation of CD11b and how CD11b influences the phenotypical and functional changes of AM, leading to the regulation of lung inflammation. Using large-scale proteomics, transcriptomics, and functional assays, we observed substantial molecular adaptations of AM during *S. pneumoniae* lung infection. Among others, comparative transcriptome and proteome analyses unraveled CD11b as a regulatory hub in AM, influencing AM plasticity, activation, and regulation of neutrophil recruitment during *S. pneumoniae* infection.

## Materials and methods

### Animals

Mice were bred and housed under specific pathogen-free conditions in the central animal facility at the University Hospital Essen and were allowed to eat and drink *ad libitum*. C57BL/6 mice (WT), 8–12 weeks of age, from Charles River, were used. The animal experiments were approved by the local animal review board of the government (Bezirksregierung Köln, Landesamt für Natur, Umwelt und Verbraucherschutz NRW in Recklinghausen, Germany), 81-02.04.2017.A470. Experimental mice were euthanized with cervical dislocation under isoflurane anesthesia for the isolation of organs.

### Culture of *S. pneumoniae* for intratracheal infection experiments

For bacterial lung infection, a pneumococcal serotype 1 strain (*S. pneumoniae* SV1, ATCC 33400) was used. *S. pneumoniae* were grown overnight on Columbia blood agar plates (Oxoid, PB5039A) at 37°C, single colonies were resuspended and cultured in 10 ml Brain–Heart Infusion Broth (Thermo Fisher Scientific, Im Steingrund 4-6, 63303 Dreieich, Germany) to mid-logarithmic phase (OD600 = 0.045–0.055; NanoDrop 1000), and 800 µl of culture was frozen at −80°C with 200 µl of 86% glycerol. For infection, bacteria were cultured to mid-logarithmic phase, centrifuged at 1,500×*g* for 10 min at 4°C, and resuspended in 550 µl PBS; 50 µl per animal corresponding to 1 × 10^8^ CFU *S. pneumoniae* was used for infection.

### Intratracheal infection with *S. pneumoniae*

Mice were anesthetized with ketamine/xylazine intraperitoneal injection at 80 mg/kg and 10 mg/kg body weight, respectively. To perform intratracheal instillation, the mouse was laid on a 60° angled self-made stand and secured by a Velcro. The teeth of the mouse were suspended on a thin rubber band, and the tongue was pulled out. Using a small-animal laryngoscope (73-4867, Harvard Apparatus, 84 October Hill Road, Holliston, Massachusetts 01746, United States), a 22-G cannula with a blunted end was inserted into the mouse trachea. The needle was removed, and 50 µl of the liquid was pipetted into the cannula. MiniVent 845 (73-4867, Harvard Apparatus) was connected to the cannula to evenly spread the liquid throughout the lungs and ventilate the lungs.

### Bronchoalveolar lavage

For bronchoalveolar lavage (BAL) extraction, the fur was wetted with ethanol and dissected to expose the rib cage. The diaphragm and lower part of the rib cage were removed to visualize the lung. The trachea was exposed by the removal of salivary glands and adventitia and cannulated with a 22-G needle (B. Braun Melsungen AG, BRAUN-4254090B). Ice-cold PBS containing 2 mM EDTA was injected with a 1-ml syringe. The first lung lavage was performed with 0.7 ml and three subsequent washes with 0.5 ml of buffer. Cell-free supernatant from the first lavage was used for the assessment of soluble molecules by ELISA, and cell pellets were combined from all four washes and used to analyze the cellular compartment by flow cytometry. The determination of BAL fluid CXCL1 was carried out using the Multiplex assay from Bio-Rad Laboratories GmbH, Kapellenstr. 12, 85622 Feldkirchen, Germany (171G5018M) according to the manufacturer’s protocol. Total protein content in the BAL was determined using Pierce™ BCA Protein Assay Kit (#23225) following the manufacturer’s protocol.

### Dissociation of murine lung into single cells

To obtain a single-cell suspension of murine lungs, the fur was wetted with ethanol and dissected to expose the rib cage. The diaphragm was removed, and the whole lung was cut out and separated from the heart. Lungs were disaggregated with 3 ml of PBS supplemented with 0.5 mg/ml collagenase D and 0.2 mg/ml DNAse I and placed in a six-well plate. Each lobe was injected with the digestion medium using a 26-G needle to facilitate the diffusion of the medium through the tissue. After 5 min of incubation at RT, the lung was disrupted with fine-toothed forceps and transferred into 5 ml polystyrene tubes, which were incubated in a water bath for 30 min. The tissue was resuspended every 10 min to mechanically boost the digestion process. The resulting single-cell suspension was filtered (100 µm), pelleted for 5 min at 4°C and 400×*g*, and processed for flow cytometry.

### Flow cytometry and cell sorting

#### Extracellular staining

The single-cell suspension (1/10 of the total lung) was liberated from erythrocytes using RCB buffer (ammonium chloride: 146 mmol, EDTA disodium: 0.2 mmol, sodium bicarbonate: 24 mmol). Antibodies were mixed in a solution of human immunoglobulins (*c* = 1.5 mg/ml, PZN-01700625, CSL Behring GmbH, Emil-von-Behring-Straße 76, 35041 Marburg, Germany) and 20 μl was added onto cell pellets, followed by a 20-min incubation at 4°C in the dark. Either 0.05 µg or 0.1 µg of each antibody were used per sample. Unbound antibodies were washed away, and the cell pellets were resuspended in PBS with 2 mM EDTA and 2% FCS (FACS buffer) for subsequent measurement on the BD LSR Fortessa II. Ten thousand Calibrite™ APC beads (BD Biosciences, Tullastraße 8-12, 69126 Heidelberg, Germany, 340487) were added to each sample to extrapolate the total cell number in the lung. BD Aria III was used for sorting AM for transcriptome and proteome analyses. Briefly, AM were identified as CD45^+^, CD11c^+^, and Siglec-F^+^ cells, whereas neutrophils were delineated based on CD45, CD11b, and Ly6G expression.

#### Intracellular staining

Intracellular staining was carried out after the staining of extracellular antigens, followed by fixation with 4% paraformaldehyde (PFA). Briefly, the cell pellet was resuspended in 100 µl of 4% PFA and incubated at 4°C for 10 min. Subsequently, cells were washed with 2 ml FACS buffer, followed by a wash with 2 ml of BD Perm/Wash (554723, BD Biosciences). A 15-min incubation step with 1 ml of perm/wash was carried out to transiently permeabilize the cells. After that, anti-mouse MPO (Hycult biotechnology b.v., Frontstraat 2A, 5405 PB Uden, Netherlands, HM1051BT) was added at 0.1 µg per sample and incubated for 30 min at 4°C in the dark. Next, Streptavidin Alexa Fluor 647 (Invitrogen is part of Thermo Fisher Scientific, Im Steingrund 4-6, 63303 Dreieich, Germany, S21374) was added at 1 µg per sample and incubated for 30 min at 4°C in the dark. The final washing step was carried out with 1 ml perm/wash, and the samples were redissolved in FACS buffer for the acquisition.

### Reactive oxygen species detection

AM reactive oxygen species (ROS) production was determined by flow cytometry using the CellROX kit from molecular probes (Thermo Fisher Scientific, C10491) according to the manufacturer’s protocol. Briefly, 3 × 10^5^ cells from the lung single-cell digests were resuspended in 100 µl of complete RPMI 1640 and incubated with 1 µM CellROX Deep red dye for 30 min at 37°C. Excess dye was washed away with 1 ml PBS, and cells were subsequently stained for extracellular markers.

### Preparation of AM for LC-MS/MS proteome

A detailed description of sample preparation, protein detection, and analysis can be found in ref (14). In brief, AM (1 × 10^5^) was lysed in 50 mM ammonium bicarbonate buffer containing 0.1% RapiGest Surfactant. The samples were reduced with 20 mM dithiothreitol for 30 min at 60°C and subsequently alkylated with 15 mM iodoacetamide for 30 min at room temperature, protected from light. Proteins were digested using 22.5 ng of trypsin per sample overnight at 37°C. The digestion was stopped by adding 0.5% trifluoroacetic acid, and the precipitated RapiGest was removed by centrifugation. Peptides were dried in vacuo, and 350 ng of peptides in 15 µl of 0.1% trifluoroacetic acid was subjected to an Orbitrap Elite mass spectrometer coupled to an Ultimate 3000 RSLCnano system. The peptides were preconcentrated for 7 min on a trap column (Acclaim PepMap 100, 300 µm × 5 mm, C18, 100 Å, flow rate: 30 µl/min) and subsequently separated on an analytical column (Acclaim PepMap RSLC, 75 µm × 50 µm, nano Viper, C18, 2 µm, 100 Å) by a gradient from 5% to 40% solvent B over 98 min (flow rate: 400 nl/min; column oven temperature: 60°C). Full-scan MS spectra were acquired by data-dependent acquisition (DDA) in the Orbitrap analyzer. Tandem spectra were measured in the linear ion trap following peptide fragmentation by collision-induced dissociation.

### Protein identification and data analysis

Peptides were identified using the Proteome Discoverer Software (v.1.4.1.14). The mass spectra were searched against the UniProtKB/Swiss-Prot database (release 2016_05, 551,193 entries) restricted to *Mus musculus* using the Mascot search engine (v.2.5). The mass tolerance was set to 5 ppm for precursor ions and 0.4 Da for fragment ions. One tryptic miscleavage was considered as well as chemical modifications of methionine (oxidation) and cysteine (propionamide). The percolator function, implemented in the proteome discoverer, was used to estimate peptide confidence, and only peptides that passed a false discovery rate (FDR) of<1% (FDR-adjusted *p*< 0.01) were considered for analysis. Ion intensity-based label-free quantification was performed using Progenesis QI for proteomics. To account for retention time shifts, LC-MS runs were aligned to one run automatically chosen by the software. A master list of features considering retention time and m/z was generated considering peptide ions with a minimum three isotopic peaks and charges state of +2, +3, and +4. The peptide identifications (peptide spectrum matches) from Proteome Discoverer were imported into the software and matched to the respective features. The protein abundances were calculated considering the normalized ion intensities of all nonconflicting peptides of a protein. Normalized protein intensities were further analyzed using bioinformatics and statistical analysis.

### AM transcriptome

AM (1 × 10^5^) were sorted using BD FACS Aria III in 100 µl of RLT lysis buffer (Qiagen GmbH, QIAGEN Straße 1, 40724 Hilden, Germany, 79216), and RNA was extracted using RNeasy Micro Kit (Qiagen, 74004) according to the manufacturer’s protocol. Using the Affymetrix IVT Pico protocol, 10 ng of total RNA was prepared and then analyzed on the Clariom S mouse array (Thermo Fisher Scientific, 902971). Hybridization, washing, and staining of the arrays were done on the GC Scanner 3000. RNA signal summarization and analysis of differentially expressed genes were performed using Partek GS v6.6 software.

### Bioinformatics and statistical analysis

Normality was tested with the Shapiro–Wilk test and homogeneity of variance with Levene’s test. For the log2 FC computation, $\text{FC}={\text{log}}_{2}\left(\frac{\bar{x_{1}}}{\bar{x_{2}}}\right)$ , was used, where $\bar{x_{1}}$ and $\bar{x_{2}}$ are the mean values of the conditions. SNR was calculated using $\text{SNR}=\left(\frac{\bar{x_{1}}-\bar{x_{2}}}{d_{1}+d_{2}}\right)$ with *d*1 and *d*2 representing the respective standard deviations. For normally distributed data, *p*-values were determined using Student’s *t*-test or Welch’s *t*-test depending on the homogeneity of variance, and for nonnormally distributed data, the Mann–Whitney *U* test was used. Subsequently, the *p*-values were adjusted for the FDR with Benjamini–Hochberg. Significant regulation was considered for the proteins and transcripts with log2 FC > ± 1 and *p*-value< 0.05.

Overrepresentation (disease ontology semantic enrichment DOSE_v.3.16.0, DO.db_v.2.9, only molecules with an absolute SNR of >2.8 were included), gene set enrichment (GSEA4.0.3, http://download.baderlab.org/EM_Genesets/July_01_2020/Mouse/UniProt/Mouse_GO_AllPathways_with_GO_iea_July_01_2020_UniProt.gmt), and Cytoscape (Cytoscape_v3.8.0, ClueGO v2.5.7, gene ontology databases from 05 August 2020, min GO level = 3, max GO level = 8, number of genes = 3, min percentage = 3.0, Kappa score threshold = 0.4) were used for functional enrichment analysis. The normalized enrichment score (NES) was calculated according to a previously published algorithm (15). The normal distribution was tested with the D’Agostino–Pearson omnibus normality test. Conditions were statistically compared by Mann–Whitney *U* test (two groups) or Kruskal–Wallis test (more than groups). A two-way analysis of variance was applied for the comparison of multiple parameters (i.e., time and genotype of mice).

Heatmaps were generated in RStudio with the packages gplots (3.1.1) and circlize (0.4.14).

### Data availability

Processed transcriptome and proteome data are available as supplementary tables. The mass spectrometry data have been deposited to the ProteomeXchange Consortium via the PRIDE partner repository with the dataset identifier PXD035269. The transcriptome data have been deposited via the NCBI partner repository with the GEO submission (GSE213547) and the NCBI tracking system #23319940.

## Results

### Lung infection by *S. pneumoniae* profoundly changes the proteome of AM

**Figure 1:**
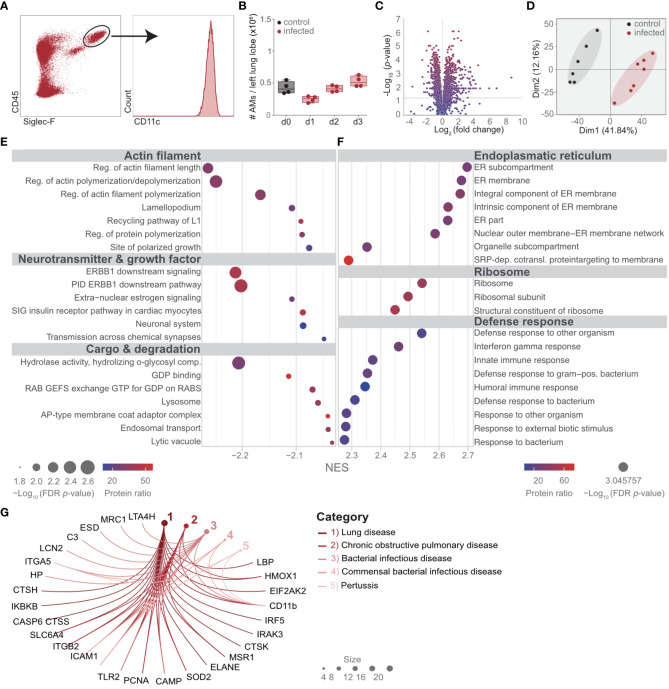
*Streptococcus pneumoniae* lung infection profoundly changes the proteome of AM. Mice were infected with 10^8^ CFUs of *S. pneumoniae* and killed on the days indicated **(B)** or on day 2 (d2) postinfection **(A, C–G)**. **(A)** The gating strategy of AM for quantitative analysis **(B)** and for cell-sorting (further details in **Supplementary Figure S1**). **(B)** Quantitative longitudinal analysis of AM after *S. pneumoniae* infection by flow cytometry. **(C, D)** AM were isolated by cell sorting as indicated in **(A)**, and the proteome was determined by LC-MS/MS. Statistical analysis of the AM proteome in control versus d2 postinfection visualized by the volcano plot. **(D)** PCA of the AM proteome in control versus day 2 after *S. pneumoniae* infection. **(E, F)** Gene set enrichment analysis of the proteome of AM to identify protein classes and pathways significantly altered on d2 postinfection compared to control mice. **(G)** Disease ontology analysis of AM proteome at day 2 after infection; shown are the pathways that contained CD11b. Molecules with an absolute signal-to-noise ratio of >2.8 were included. The data are from min to max with a line indicating the median and a box extending from the 25th to 75th percentiles, and the Kruskal–Wallis test for comparison of multiple groups was used **(B)**. *n* = 7 (control) and *n* = 6 (infected) **(A, C–G)** or indicated by the number of points **(B)**.

To study the proteomic changes in AM upon bacterial lung infection, mice were intratracheally infected with *S. pneumoniae*, and the number of CD45^+^, Siglec-F^+^, and CD11c^+^ AM was determined until day 3 postinfection (**Figures 1A, B**; **Supplementary Figure S1**). A slight reduction in AM was observed on day 1, and the population recovered by day 3 postinfection (**Figure 1B**). To delineate the molecular adaptations of AM upon lung infection, AM was isolated by cell sorting from lung digests on day 2 after infection and from control mice (**Supplementary Figure S1**). Proteins were extracted and processed for measurements by DDA LC-MS/MS. In total, 3,607 proteins were detected, of which 502 proteins were significantly upregulated and 836 were significantly downregulated (**Figure 1C**; **Supplementary Table S1**). Dimensionality reduction by a principal component analysis (PCA) indicated a strong separation of control and infected AM, with a high degree in the first component of 41.84% (**Figure 1D**; **Supplementary Table S1**). Gene set enrichment analysis (GSEA), which assigns classes of proteins to specific functional pathways, indicated reduced expression of molecules of the *actin filament*, *neurotransmitters*, and *growth factors* as well as *cargo* and *degradation* (**Figure 1E**; **Supplementary Tables S1**, **S2**). In contrast, proteins associated with the *endoplasmic reticulum*, the *ribosome*, and the *defense response* were significantly enriched (**Figure 1F**; **Supplementary Table S3**). Furthermore, disease ontology analysis (DOSE) indicated that the altered proteome of AM was associated with lung-specific diseases. Among others, DOSE indicated the diseases “lung diseases” and “*COPD*”, and interestingly, CD11b was curated to those disease ontologies (**Figure 1G**; **Supplementary Table S4**).

These data indicate a strong proteomic switch in AM, which might be involved in the regulation of the immune response during lung infection with *S. pneumoniae.*

### AM express neutrophil-associated molecules upon lung infection on a protein level

**Figure 2:**
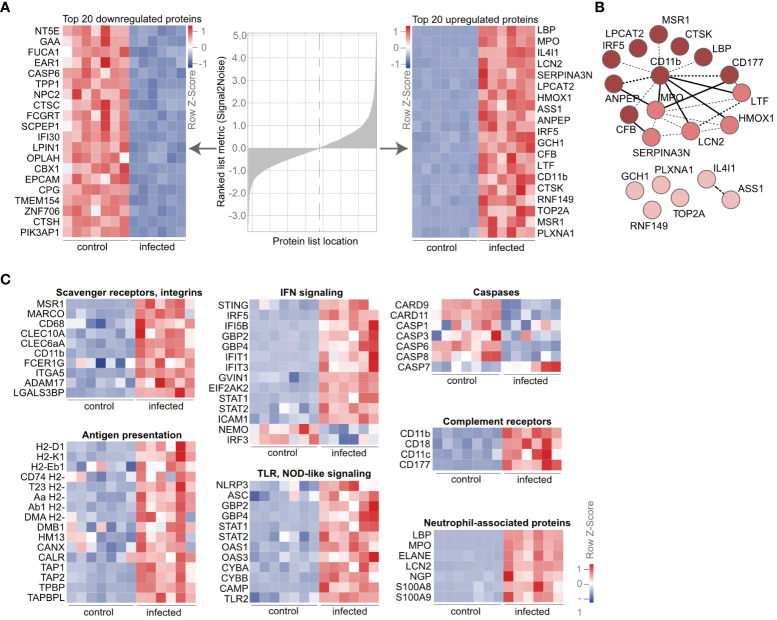
AM express neutrophil-associated proteins during lung infection. **(A)** Mice were infected with *S. pneumoniae*, and AM from control and infected mice were isolated by cell sorting as indicated in **Figure 1A**. The proteome was determined by LC-MS/MS, and the heat map of the most down- and upregulated proteins at day 2 post-*S. pneumoniae* infections were ranked by signal-to-noise ratio. **(B)** STRING network of the top 20 upregulated proteins from **(A)** (https://version-11-0b.string-db.org/cgi/network?networkId=bxjxswWZpYme). The color code indicates clusters based on *k*-means (*k* = 3), while dashed lines indicate edges within one cluster and solid lines between different clusters. **(C)** Heat map of regulated molecules and pathways upon infection. Order in the subclusters according to fold change. **(A–C)** *n* = 7 (control), *n* = 6 (infected). IFN, interferon; NOD, nucleotide-binding oligomerization domain; TLR, Toll-like receptor.

To identify the most differentially expressed proteins in AM upon infection, we calculated the signal-to-noise ratio of proteins in AM from infected versus control mice (**Figure 2A**). Among the top downregulated proteins, we found immunosuppressive molecules such as NT5E, a surface enzyme that hydrolyses the danger signals ATP and ADP into anti-inflammatory adenosine. Furthermore, cysteine protease CASP6, which regulates cell death, and lysosomal enzymes involved in carbohydrate (GAA, FUCA1) and lipid metabolism (NCP2, LPIN1) were strongly downregulated. On the other hand, the phagocytic receptor LBP, myeloperoxidase (MPO), the antimicrobial protein lipocalin (LCN2), the proinflammatory transcription factor IRF5, complement protein CFB and complement receptor 3, CD11b, as well as proteins in the NO generation pathway, e.g., ASS1, GCH1, and MSR1A, were among the 20 most overexpressed proteins (**Figure 2A**). Interestingly, the DNA topoisomerase TOP2A was also strongly upregulated, suggesting *in situ* proliferation of AM, accounting for the recovery after the slight decrease on day 2 postinfection (**Figure 1B**). Using the strongly enriched molecules upon infection, we performed a protein–protein interaction analysis through STRING. Such enrichment analysis identified CD11b with the most interaction partners among the top 20 enriched molecules (**Figure 2B**). Detailed analysis of individual proteins of the proteomic dataset revealed increased expression of molecules associated with “scavenger receptors” and “integrins” (**Figure 2C**; **Supplementary Table S1**). We observed increased expression of the pattern recognition receptors MSR1, MARCO, CD68, and ITGA5, as well as C-type lectins, CLEC10A (MGL, CD301a), and CLEC6A (dectin-2), and complement receptors capable of recognizing necrotic cells by complement-mediated phagocytosis, such as CR3 (CD11b/CD18) and CR4 (CD11c/CD18). We found that the lung infection drives AM to upregulate proteins of the “antigen presentation” machinery, such as class I and class II histocompatibility molecules MHCI and MHCII, calnexin (CANX), calreticulin (CALR), and transporters associated with antigen processing (TAP1, TAP2), suggesting activation and phagocytosis of foreign antigens. The activated phenotype of AM was further evident by proteins of the “IFN signaling” cascade, “TLR signaling”, and downregulation of several “caspases”. In addition to CD11b, several proteins are known to be strongly expressed by neutrophils, such as myeloperoxidase (MPO), the antimicrobial protein lipocalin (LCN2), neutrophil gelatinase-associated lipocalin (NGAL), and neutrophil elastase (ELANE), were strongly enriched in AM upon infection (**Figure 2C**; **Supplementary Table S1**). These data indicate specific proteomic changes in AM upon infection and suggest an activated phenotype of AM with phagocytic activity.

### AM recruit and phagocytose neutrophils upon lung infection with *S. pneumoniae*

**Figure 3:**
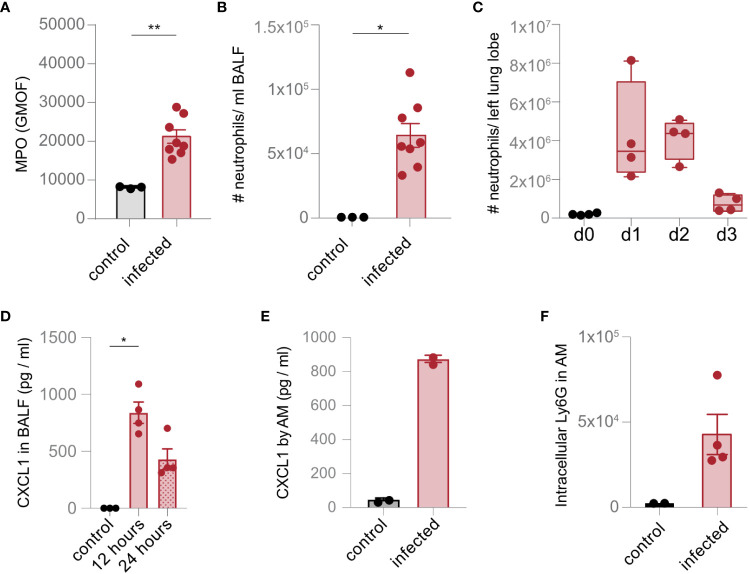
AM recruit and phagocytose neutrophils upon lung infection with *S. pneumoniae*. **(A)** The level of MPO was measured in the lung in control mice and 1 day after infection (infected) by flow cytometry. **(B, C)** The number of Ly6G^+^ neutrophils in the BALF **(B)** and the lung **(C)** were analyzed by flow cytometry (gating scheme in **Supplementary Figure S2**) 1 day after infection **(B)** and at the time points indicated **(C)**. **(D)** The expression of CXCL1 was determined by a multiplex assay 1 day after infection. **(E)** AM were isolated by cell sorting (gating scheme in **Figure 1A**). Cells were left unstimulated (control) or incubated with *S. pneumoniae* (infection). The expression of CXCL1 was evaluated by flow cytometry 24 h after stimulation. **(F)** Phagocytosis of neutrophils by AM was determined *ex vivo* by intracellular staining of Ly6G in AM on day 2 after *S. pneumoniae* infection by flow cytometry. The data are from min to max with a line indicating the median and a box extending from the 25th to 75th percentiles **(C)** or mean ± SEM **(A, B, D–F)**; ^*^ *p<* 0.05; ^**^ *p<* 0.01, Kruskal–Wallis test **(C, D)**, and Mann–Whitney *U* test **(A, B, E, F)** were used. *n* = indicated by the number of points.

We hypothesized that the accumulation of neutrophil-associated proteins in AM and the upregulation of scavenger and complement receptors were consequences of neutrophil recruitment and subsequent phagocytosis. Indeed, strong expression of MPO was detected in the infected lung, and a high number of neutrophils were recruited into the BALF and the lung tissue upon infection (**Figures 3A–C**; **Supplementary Figure S2**). Moreover, we observed stronger expression of CXCL1 in the BALF and by AM (**Figures 3D, E**). Increased abundance of neutrophils in the lung was also associated with intracellular expression of the neutrophil marker Ly6G in AM (**Figure 3F**), suggesting recruitment and phagocytosis of neutrophils through AM during lung infection with *S. pneumoniae*.

### Transcriptomic analysis reveals increased CD11b transcript and ROS production by AM upon *S. pneumoniae* infection

**Figure 4:**
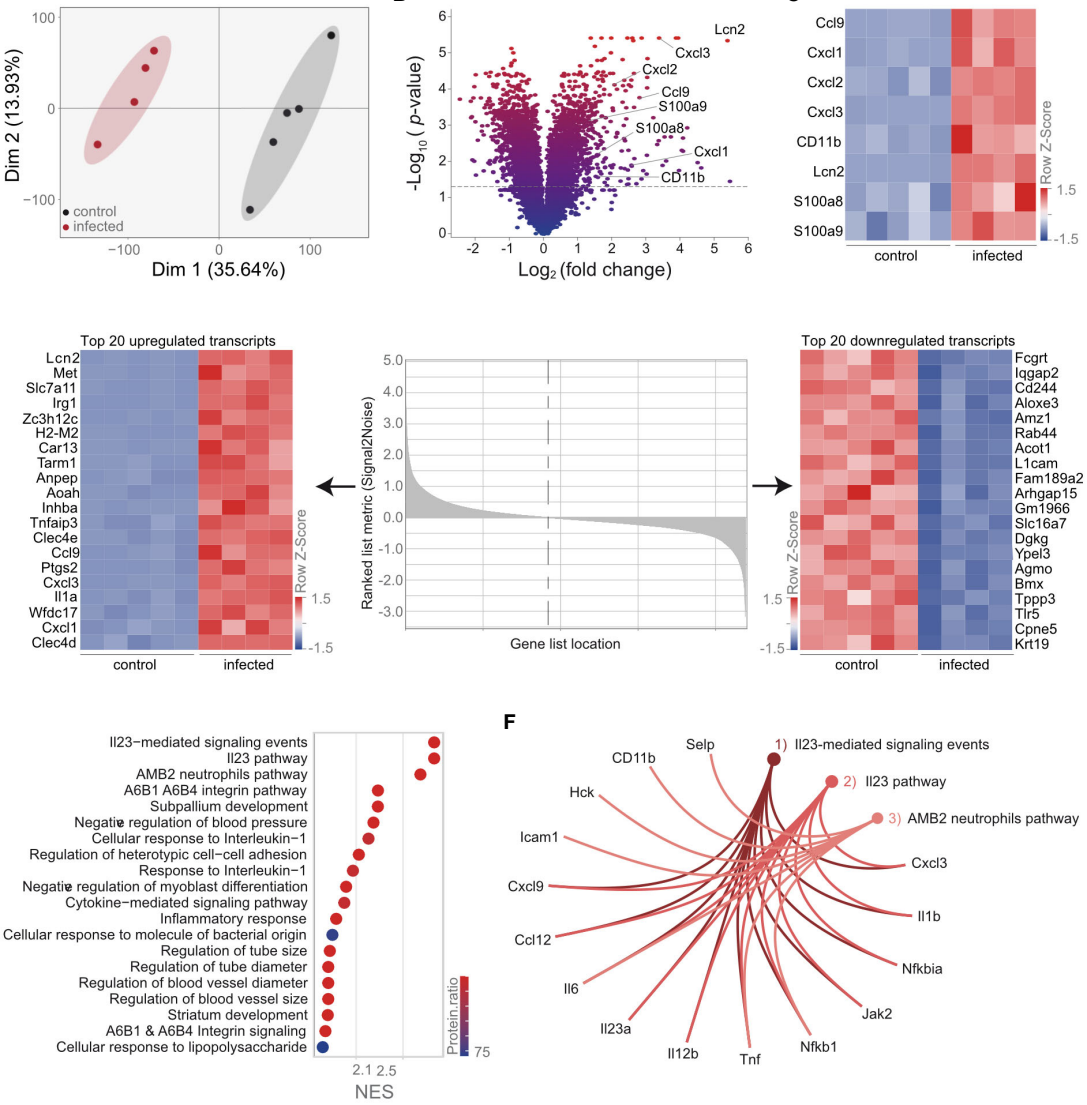
Transcriptional analysis of AM reveals an activated phenotype and upregulation of the IL-23 pathway. Mice were infected with *S. pneumoniae.* AM were isolated 12 h postinfection from control and infected mice by cell sorting (gating scheme in **Figure 1A**) for transcriptomic analysis. **(A)** PCA of the AM transcriptome of control and infected mice. **(B)** Statistical analysis of the AM transcriptome in control and infected mice indicated by the volcano plot. **(C)** Expression of molecules in the transcriptome data, which are associated with neutrophil recruitment. **(D)** Heat map of the top 20 down- and upregulated AM transcripts post-*S. pneumoniae* infection ranked by signal-to-noise ratio. **(E)** GSEA of AM transcriptome after infection. **(F)** Visualization of the most enriched pathways in the transcriptome after GSEA. **(A–F)** *n* = 5 (control), *n* = 4 (infected).

Our data indicate that the proteome of AM is strongly influenced by the phagocytosis of neutrophils. To provide further information on the cell-specific molecular adaptations of AM upon *S. pneumoniae* lung infection and to analyze specific molecules, i.e., *Cd11b*, on a transcriptional level, we measured the transcriptome of cell-sorted AM. Principal component analysis indicated pronounced transcriptomic changes in AM upon infection (**Figure 4A**; **Supplementary Table S5**). Among others, we observed strong upregulation of molecules involved in neutrophil recruitment, such as *Ccl9*, *Cxcl1*, *Cxcl2*, *Cxcl3*, *Cd11b*, *Lcn2*, *S100a8*, and *S100a9*, as well as increased expression of *Cd11b* (**Figures 4B, C**; **Supplementary Table S5**). Moreover, *Cxcl1*, *Cxcl3*, *Ccl9*, and *Lcn2* were among the most significantly regulated (**Figure 4D**; **Supplementary Table S5**). Notably, gene set enrichment analysis indicated strong upregulation of the IL-23-related pathway (**Figure 4E**; **Supplementary Table S6**), characterized by increased expression of the molecules that polarize and recruit neutrophils, such as *Cxcl3*, *Il1b*, *Il6*, *Il12b*, *Il23a*, and *Tnfα* (**Figure 4F**; **Supplementary Table S6**). These data confirm that AM is directly involved in the recruitment of neutrophils.

### CD11b by AM constitutes a central molecule in the cell-specific protein and transcript network

**Figure 5:**
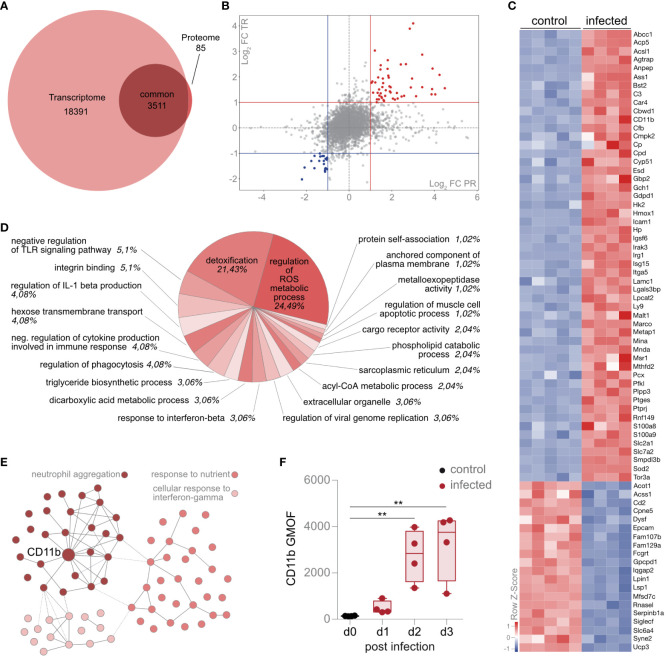
Intrinsic upregulation of CD11b by AM regulates neutrophil aggregation, metabolism, and activation. **(A)** Mice were infected with *S. pneumoniae*, and AM from control and infected mice were sorted for transcriptome (12 h postinfection) and proteome analysis (d2 postinfection). Intersection of AM-specific proteome and transcriptome data in a Venn plot. **(B)** Correlation analysis of transcript (TR) and protein (PR) fold changes (FC). FC, fold change; PR, proteome, TR, transcriptome. **(C)** Heat map f the top 73 down- and upregulated molecules on a transcript level, indicated as *Z*-scores. **(D)** Functional enrichment analysis of the 73 molecules (up- and downregulated) indicated in **(C)** by Cytoscape and Cluego (Cytoscape settings in the Materials and methods section). The percentage indicates the proportion of molecules detected per pathway. **(E)** Protein–protein interaction analysis of the 73 molecules coregulated in proteome and transcriptome by STRING interaction network (*k*-means clustering: *k* = 3) (https://version-11-5.string-db.org/cgi/network?networkId=bXTD8booQYHB). **(F)** Flow cytometry analysis of CD11b expression by AM during *S. pneumoniae* infection. The data are min to max with a line indicating the median and a box extending from the 25th to 75th percentiles **(F)**; ^**^ *p<* 0.01, Kruskal–Wallis test **(F)** was used. **(A–E)** *n* = 5 (control), *n* = 4 (infected) for transcriptome and *n* = 7 (control), *n* = 6 (infected) for proteome. **(F)** *n* = indicated by the number of points.

To compare the transcriptional and proteomic adaptations of AM, we analyzed the 3,511 molecules that were measured by transcriptomics and proteomics (**Figure 5A**). Correlation analysis identified 73 coregulated molecules based on transcriptional and proteomic log_2_ fold changes of > ± 1 (**Figure 5B**; **Supplementary Table S7**). Among others, the alarmins *S100a8* and *S100a9* were upregulated. Furthermore, CD11b, which is responsible for cell communication, migration, and adhesion, was also upregulated on a transcriptomic and proteomic level (**Figure 5C**; **Supplementary Table S7**). Enrichment analysis of the 73 most differentially regulated molecules by STRING provided insights into the global molecular changes of AM upon infection. We detected the pathways “Regulation of ROS metabolic process” and “Detoxification”, which accounted for the majority of coregulated molecules on a transcript and proteome level (**Figure 5D**). Since ROS production correlates with the phagocytotic activity of AM, we isolated AM upon infection and validated ROS expression by a flow cytometric assay (**Supplementary Figure S2**). Next, we used STRING to indicate molecular interactions between the molecules, aimed at identifying molecular hubs in AM upon infection. We identified three individual clusters by *k*-means (**Figure 5E**; **Supplementary Figure S3**; **Supplementary Table S8**). The proteins in cluster 1 (light red) suggested an enrichment of the “Cellular response to interferon-gamma” (**Figure 5E**; **Supplementary Figure S3**; **Supplementary Table S9**), and proteins in cluster 2 (mid-red) were associated with “Response to nutrient” (**Figure 5E**; **Supplementary Figure S3**; **Supplementary Table S10**). Proteins in cluster 3 (dark red) were functionally enriched in the processes of *neutrophil aggregation* (**Figure 5E**; **Supplementary Figure S3**; **Supplementary Table S11**). Using this protein–protein network analysis, CD11b showed the most interactions in this k-means-based interaction map (**Figure 5E**), confirming a central role of this integrin in AM response upon lung infection with *S. pneumoniae* and the involvement of neutrophil activation and migration.

Next, we validated the increased expression of CD11b on AM by antibody-based staining and subsequent flow cytometry. The expression of CD11b was significantly upregulated in AM during infection (**Figure 5F**). Thus, AM regulates CD11b in response to a lung infection with *S. pneumoniae* on a transcriptomic and proteomic level, and cluster analysis identifies CD11b as a central molecule regulating the neutrophil-associated antimicrobial defense.

## Discussion

AM shapes the immune response in the lung during bacterial infections. These cells encounter respiratory pathogens and are critically involved in the regulation of inflammation through the recruitment and clearance of leukocytes. We detected an effective antimicrobial response of AM upon infection with *S. pneumoniae* through the secretion of molecules to recruit neutrophils as well as effective phagocytosis of neutrophils. We also observed significant molecular adaptations on a transcriptomic and proteomic level by AM upon infection with *S. pneumoniae*. Among others, AM strongly upregulated the integrin CD11b during the disease. Our study reveals cell-specific transcriptomic and proteomic adaptations of AM during infection with *S. pneumoniae* that critically influenced the antimicrobial response.

We observed an inflammatory functionality of AM during the disease, characterized by increased metabolism, changes in expression of the integrin CD11b, expression of chemokines to recruit neutrophils, and transcriptional regulation of *Il1b*, *Il6*, *Il12b*, *Il23a*, and *Tnfα*. We also observed activation of the complement pathway as well as proteins ASS1 and GCH1, which regulate the generation of nitric oxide (16, 17). On the other hand, anti-inflammatory molecules were concomitantly induced in AM, such as IL-4I1, which is involved in macrophage polarization and can directly influence nitric oxide generation through the degradation of l-arginine (18). We also observed the upregulation of several receptors that are important for sensing the tissue microenvironment (19). Among others, C-type lectins, such as CLEC10A, a macrophage glycoreceptor for the uptake of pathogens and damaged cells, were upregulated upon infection (20). Another C-type lectin, CLEC6A, also showed increased expression, which might lead to phosphorylation of the immunoreceptor tyrosine-based activation motif (ITAM) of the F_c_e receptor, triggering activation of transcription factor NF-κB and production of TNF-α and IL-1 receptor antagonists (21, 22). Overall, these manifold changes in the population of AM may be mediated by various mechanisms, specifically by leukocyte recruitment. In particular, monocytes that can differentiate into monocyte-derived AM (mo-AM) may play a dominant role (23). This recruitment of mo-AM alters the population of tissue-resident (TR) AM (24, 25) and represents an important mechanism for the phenotypic and functional adaptation of AM during infection. Our study does not distinguish between different subpopulations of AM. However, our sorting strategy was based on Siglec^hi^-expressing AM, assuming that the proteomic and transcriptomic changes refer to the pool of TR-AM (23).

We also observed the expression of *Il23*, a key proinflammatory cytokine, produced by activated macrophages (26). Excess of *Il23* has pathological consequences by facilitating chronic inflammation and tissue damage (27). *Il23* signaling facilitates the production of proinflammatory mediators and the differentiation of TH17 cells and thereby promotes the recruitment, polarization, and activation of neutrophils, causing tissue damage and inflammation (28, 29). We observed increased neutrophil recruitment upon infection. Among others, neutrophils are recruited through chemokines and alarmins, which were increasingly expressed on a transcript and proteome level upon infection, suggesting that AM are actively involved in neutrophil attraction. AM also phagocytosed neutrophils upon infection, indicating that they are not only involved in the recruitment but also in the resolution of inflammation.

Registration of the transcriptome and proteome data through bioinformatic analyses identified several differentially expressed molecules and pathways in *S. pneumoniae* infection. Among others, the integrin CD11b was differentially regulated in a temporal and bacterial burden-dependent manner. Moreover, protein–protein interaction analysis identified CD11b as a central regulatory hub important for AM activation after infection. It has been shown that CD11b facilitates the uptake of *S. pneumoniae* through interaction with the pilus subunit RrgA (30). Furthermore, CD11b might also be involved in the uptake of complement-coated *S. pneumoniae*, limiting bacterial growth (31). The defense response against *S. pneumoniae* involves recognition by several pattern recognition receptors (PRRs) and scavenger receptors, such as TLR2, TLR4, and MARCO (32, 33), which leads to internalization and phagosomal degradation of bacteria. Recognition of *S. pneumoniae* by cytosolic PRRs leads to the induction of type-I interferon (IFN) (34), which was also observed during our lung infection model. The regulation of both type-I IFN (35) and PRRs (36) signaling can occur through CD11b, and there is increasing evidence for CD11b in immunomodulation as well as upregulation of AM in humans and mice (11).

In summary, our detailed molecular and functional analysis indicated critical AM-specific adaptions in *S. pneumoniae* infections. We reveal transcriptomic and proteomic adaptations of AM during lung infection with *S. pneumoniae*, regulation of the integrin CD11b, and identification of profound molecular alterations in the AM compartment that are critical for effective antimicrobial immunity and regulation of inflammation.

## Data availability statement

The datasets presented in this study can be found in online repositories. The names of the repository/repositories and accession number(s) can be found in the article/**Supplementary Material**.

## Ethics statement

The animal study was approved by LANUV, Recklinghausen, Germany. The study was conducted in accordance with the local legislation and institutional requirements.

## Author contributions

The experiments were performed by DK, DVT, JB, KZ, NK, NS, RC, and ST. Mass spectrometry and transcriptomic measurements, data analysis, visualization, and computational analysis were performed by BB, BS, DS, JJ, LK-H, ML, OS, PS, and TB. Critical reagents and discussion were provided by DT, FW, and VJ. The study was written by KZ, ST, OS, and DRE and supervised by DRE. All authors have read and commented on the article. All authors contributed to the article and approved the submitted version.

## Supplementary material

Supplementary Figure 1 Representative gating strategy of AM for isolation by cell-sorting and flow cytometry analysis. Related to **Figures 1A, B**, **2A–C**, **4A–F**, **5A–E**.

Supplementary Figure 2
**(A)** Representative gating strategy of neutrophils for flow cytometric analysis. Related to **Figures 3B, C**. **(B)** Analysis of ROS production in the lung one day after infection by flow cytometry.

Supplementary Figure 3 Original STRING network with all molecule names and *k*-means cluster. Related to **Figure 4E**.

Supplementary Table 1 Normalized abundance of proteins identified by LC-MS/MS and corresponding statistics. Related to **Figures 1C–G**.

Supplementary Table 2 Upregulated pathways on the proteomic level analyzed by GSEA. Related to **Figure 1F**.

Supplementary Table 3 Downregulated pathways on the proteomic level in infected versus control analyzed by GSEA. Related to **Figure 1E**.

Supplementary Table 4 Overrepresented diseases analyzed by DOSE. Related to **Figure 1G**.

Supplementary Table 5 Transcriptome analysis by Clariom S mouse array and corresponding statistics. Related to **Figures 4A–F**.

Supplementary Table 6 Pathways upregulated on the transcriptomic level in infected versus control mice analyzed by gene set enrichment analysis. Related to **Figures 4E, F**.

Supplementary Table 7 Normalized abundance and expression intensities of the mutual molecules measured by proteomics and transcriptomics and corresponding statistics. Related to **Figures 5A–C**.

Supplementary Table 8 Tabular overview of the *k*-means clusters of the 73 in proteome and transcriptome coregulated molecules and permalink to STRING. Related to **Figure 5E**.

Supplementary Table 9 GO Biological Process enriched terms of *k*-means cluster 1 with permalink. Related to **Figure 5E**.

Supplementary Table 10 GO Biological Process enriched terms of *k*-means cluster 2 with permalink. Related to **Figure 5E**.

Supplementary Table 11 GO Biological Process enriched terms of *k*-means cluster 3 with permalink. Related to **Figure 5E**.
