# Supplementary figures and images for: Comparative transcriptomic and proteomic signature of lung alveolar macrophages reveals the integrin CD11b as a regulatory hub during pneumococcal pneumonia infection
Source: Front Immunol. 2023 Sep 18;14:1227191. doi: 10.3389/fimmu.2023.1227191 (PMC10544576; doi:10.3389/fimmu.2023.1227191)

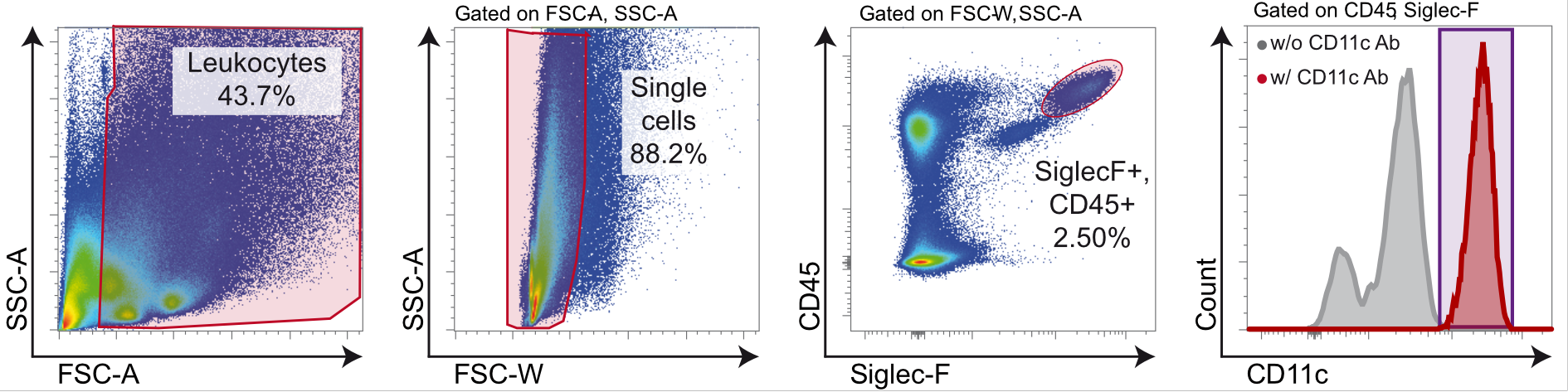

Supplement: Supplementary Figure 1 — Representative gating strategy of AM for isolation by cell-sorting and flow cytometry analysis. Related to Figures 1A, B , 2A–C , 4A–F , 5A–E . [file Image_1.tiff]

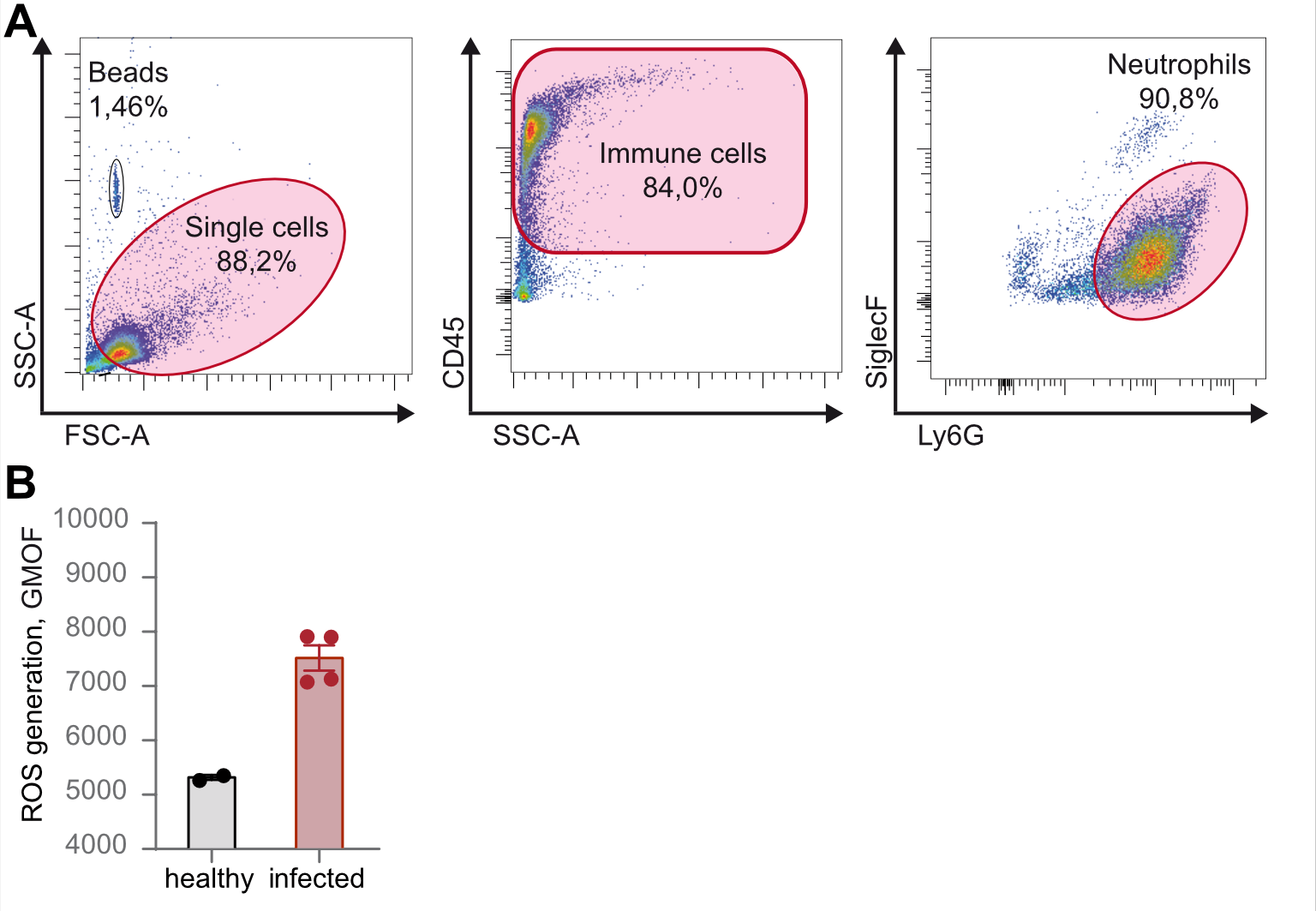

Supplement: Supplementary Figure 2 — (A) Representative gating strategy of neutrophils for flow cytometric analysis. Related to Figures 3B, C . (B) Analysis of ROS production in the lung one day after infection by flow cytometry. [file Image_2.tiff]

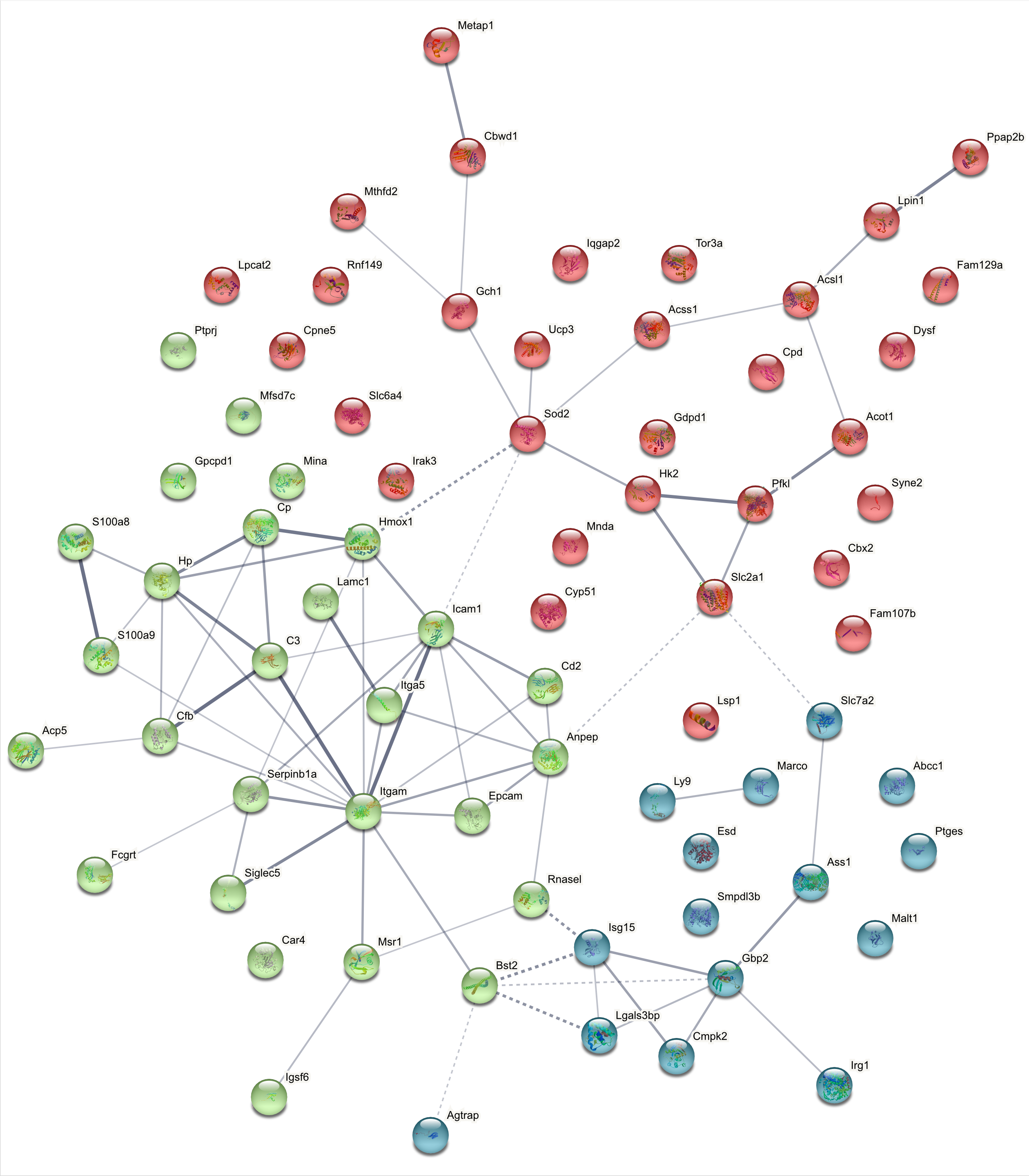

Supplement: Supplementary Figure 3 — Original STRING network with all molecule names and k-means cluster. Related to Figure 4E . [file Image_3.tiff]
